# Advancements in Low-Chill Blueberry *Vaccinium corymbosum* L. Tissue Culture Practices

**DOI:** 10.3390/plants9111624

**Published:** 2020-11-23

**Authors:** Francesco Cappai, Alexandria Garcia, Ryan Cullen, Matthew Davis, Patricio R. Munoz

**Affiliations:** Blueberry Breeding and Genomics Laboratory, Horticultural Sciences Department, University of Florida, Gainesville, FL 32611, USA; francesco.cappai@ufl.edu (F.C.); alexgarcia0102@ufl.edu (A.G.); rcullen@ufl.edu (R.C.); mattdavis9806@ufl.edu (M.D.)

**Keywords:** blueberry, plant transformation, plant hormones, light quality, selection agents

## Abstract

The demand for blueberry *Vaccinium corymbosum* L. (and hybrids) plants has significantly increased in the last 30 years due to its market expansion. In vitro propagation of sterile plants are required for commercial purposes but also for research applications such as plant transformation. Thus far, tissue culture characteristics of the tropical-adapted blueberry have been scarcely studied. In this study we present the following findings: (i) zeatin, a hormone used to promote plant growth, should be used in the 1–2 mg/L range to promote plant architecture optimal for transformation experiments; (ii) red-blue LED lights induce more production of meristems and biomass than white LED or fluorescent lights; (iii) levels as high as 1000 mg/L of decontamination agents (the antibiotics timentin and cefotaxime) can be used to eliminate *Agrobacterium* overgrowth without inhibiting plant growth during plant transformation experiments; (iv) kanamycin, paromomycin, and geneticin, which are widely used antibiotics to select transgene-carrying transformants, cannot be efficiently used in this system; (v) glufosinate, a widely used herbicide, shows potential to be used as an effective selectable marker for transformed plants.

## 1. Introduction

Blueberries are an asexually propagated (i.e., clonally) crop, traditionally achieved by rooting softwood cuttings [1,2]. However, numerous modern operations rely on in vitro propagation to meet the growing plant needs. Blueberry in vitro tissue culture allows for rapid replication via internodal propagation and is also essential for all endeavors that require sterile starting materials, such as genetic transformation and, more recently, gene editing.

Efficient internodal propagation depends on multiple factors. Some of these factors include genotype; type and concentration of plant growth hormones in the media; concentration of media components such as vitamins, salts, and elements; and incubation conditions such as temperature and light [3]. While the media composition has previously been broadly optimized [4], some factors can be genotype-specific. The use of hormones, fertilizers, and light can all be considered growth regulators that can enhance meristem proliferation, and by extension overall plant growth [5]. For biotechnological applications, decontamination agents (timentin and cefotaxime) and selection agents (e.g., kanamycin) are also crucial. These allow the operator to move the plants along the transformation pipeline, eliminating bacteria and plants that have not been transformed, respectively [6,7].

**Figure 1:**
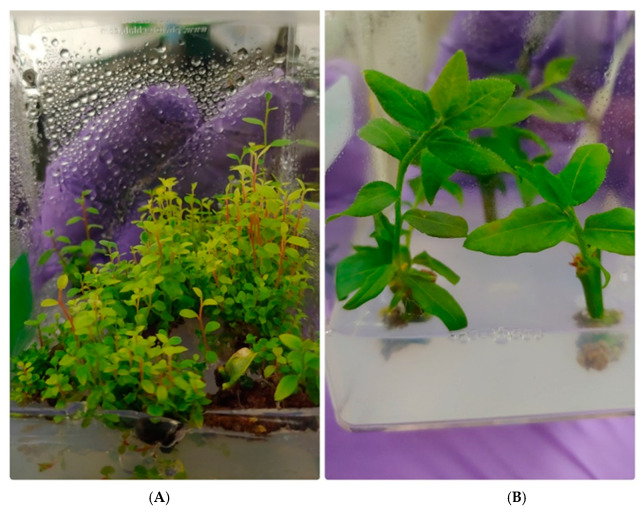
Example of different low chill highbush blueberry plant architectures after 8 weeks in tissue culture. (**A**) an architecture characterized by numerous meristems and small leaves from the cultivar “Legacy” grown on stock culture media containing 4 mg of zeatin and under RB LED lights. (**B**) an architecture characterized by a single shoot and large leaves from the cultivar “FL11-35” grown on stock culture media containing 1 mg of zeatin and under fluorescent white lights.

Artificial/synthetic cytokinins zeatin, thidiazuron (TDZ), and 2-isopentenyladenine (2iP), have been used to promote in vitro shoot formation from internodal cuttings, with multiple reports that zeatin is the most effective at shoot regeneration [8,9]. Zeatin is still widely used in High Chilling (HC) cultures [8,10], where the media concentration range varies among publications between 0.5 and 4 mg/L [8,9,11,12]. The optimal amount is not only genotype-specific, it also depends on the desired application, as cytokinins affect meristem proliferation [13]. For plant production, numerous robust nodal segments are required, while for plant transformation, fewer, larger, and easier to handle leaves (i.e., fewer nodal segments) are desirable (Figure 1) [3]. Light can also be considered a plant growth regulator as different light wavelengths, intensity and amounts can initiate intracellular signaling cascades for example causing conformational changes in the backbone of cellular receptors called phytochromes, which can then transition to their biologically active form [14,15].

Like hormones, nutrients in the media need to be balanced. Woody Plant Medium (WPM) [16] with additional Calcium Nitrate (CN) is routinely used for blueberry tissue culture [4,9]. CN is used as a fertilizer both in vivo and in vitro and it is reportedly added in the 500–800 mg/L range [4,9,17,18]. However, an early study also reports a concentration of 141.69 mg/L, although a medium other than Woody Plant Media (WPM) was used as a base in this case.

Obtaining robust and large stems and leaves are not only instrumental to micropropagation, but also to other techniques such as genetic transformation [3]. Blueberry transformation, a necessary step in gene editing, is achieved by DNA-carrying-*Agrobacterium tumefaciens* infection of mature excised leaves [4,18]. It has been shown that blueberry, a challenging crop in terms of genetic transformation, can be better infected with the hypervirulent *Agrobacterium* strain, EHA105 [19]. Although very effective at entering the plant systems, EHA105 can cause downstream challenges as its aggressiveness makes it hard to remove from the plant tissue culture once its DNA-transfer task has been fulfilled [20]. For this reason, two decontamination agents, cefotaxime and timentin, are routinely used in the later blueberry transformation stages to remove this EHA105. Cefotaxime is reportedly added to the media in the 100–500 mg/L range [18,19,21], whereas timentin is mentioned to be added at 250 mg/L [18,22].

Additionally, the successful transformation also requires the use of effective transformation markers that, when added to the media, inhibit/kill untransformed plants while allowing transformed (resistance-carrying) plants to grow [18]. Kanamycin has been used as the main selectable marker in blueberry in concentrations that range from 10 mg/L to 50 mg/L [18,19]. However, endogenous kanamycin resistance has been reported in a few crops such as sweet potato (*Ipomea batatas* L.) [23], potato (*Solanum tuberosum* L.) [24], corn (*Zea mays* L.) [25,26], and grasses [27]. Two other antibiotics, G-418 (geneticin) and paromomycin have also been used in plant transformation as substitutes for kanamycin since the gene nptII confers resistances to all three compounds [28]. In different species, geneticin is reportedly used in the 20–50 mg/L range [29,30,31], whereas paromomycin is used in the 50–400 mg/L range [32,33,34]. Even though HC blueberry was proven to be susceptible to kanamycin [18], a potential alternative has been developed using the herbicide glufosinate as a selectable marker, which is effective at 0.1 mg/L [22].

Commonly cultivated tetraploid blueberries are divided into two broad subclassifications based on the chill hours required to flower, high-chill varieties, and low-chill (LC) varieties [35]. This classification roughly corresponds to the division between northern and southern highbush hybrids, the latter ones containing sub-genomes from other *Vaccinium* species introgressed via traditional breeding [36]. Most published research is on HC blueberry cultivars, whereas physiological and molecular knowledge specific to LC cultivars is still lacking. This impacts tissue culture operations, as hormonal and antibiotic responses could differ from what is published for HC cultivars.

The study presented here investigates the best tissue culture practices for “Legacy”, a widely used HC cultivar, and “Farthing” and “Colossus”, two LC varieties are chosen for their vigor and representativeness of LC characteristics. Factors previously studied in HC blueberry and other crops are here considered: (i) zeatin dose in the media and CN for optimal growth; (ii) cefotaxime and timentin as decontamination agents, (iii) kanamycin, geneticin, paromomycin, and glufosinate as selectable markers.

## 2. Results

### 2.1. Introduction to Tissue Culture from Field Conditions

Efficient introduction to tissue culture from field conditions is critical to all downstream applications as it determines the amount and quality of the available plant materials. We employed a published “basic technique” that we improved over time and eventually elaborated an “advanced technique”. The basic technique was not very effective in preserving plant viability while removing pathogens, with average mortality rates of 89.44% and 82.76% for “Legacy” and “Farthing”, respectively. Conversely, the numerous improvements implemented in the advanced technique made a significant difference (*p* < 0.005), yielded a dramatic mortality decrease which plummeted to 7.36% and 7.04% for “Legacy” and “Farthing”, respectively.

### 2.2. Hormonal Dosage and Nutrient Optimization

**Figure 2:**
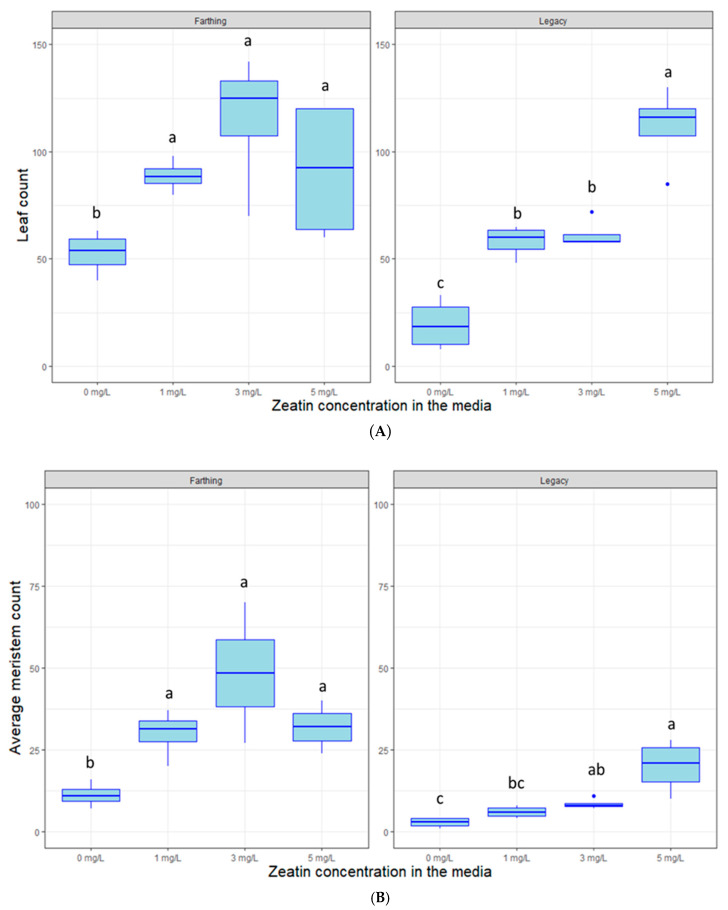
Box plot distribution of the effect of zeatin concentration on (**A**) leaf proliferation and (**B**) meristem proliferation in blueberry genotypes “Farthing” (left panels) and “Legacy” (right panels) grown in tissue culture. Letters above boxplots indicate multiple comparison test. Treatments with the same letters are not statistically different at alpha = 0.05.

The plant hormone zeatin can affect plant meristem and leaf proliferation based on its concentration and genotype that is placed in the media. In most cases, “Farthing” plant cuttings produced more stems and leaves in tissue culture than “Legacy” plant cuttings (e.g., Figure 2). As expected, the hormone zeatin affected leaf proliferation and the number of shoots on the two blueberry genotypes (Figure 2). The concentrations of zeatin increased leaf count for “Legacy” (*p* < 0.005) and “Farthing” (*p* < 0.01). Likewise, zeatin also increased meristem proliferation in “Legacy” (*p* < 0.005) and “Farthing” (*p* < 0.05). For Farthing, leaf production seemed to peak at 3 mg/L, whereas for “Legacy” the largest number of leaves was produced at 5 mg/L. Empirically, we observed the concentration of 1 mg/L yielded for both genotypes a consistent result, which was the objective of this experiment. We also observed fewer, larger leaves with 1 mg/L.

**Figure 3:**
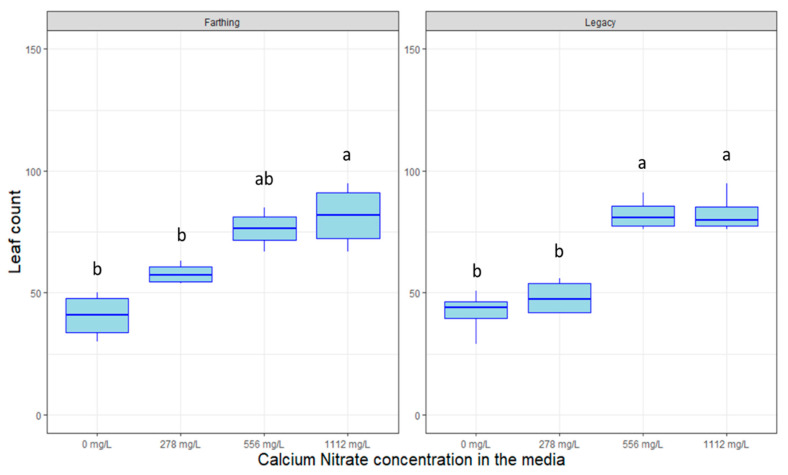
Box plot distribution of the effect of calcium nitrate concentration on leaf proliferation of blueberry genotypes “Farthing” (**left** panel) and “Legacy” (**right** panel) grown in tissue culture. Letters above boxplots indicate multiple comparison test. Treatments with the same letters are not statistically different at alpha = 0.05.

Calcium Nitrate (CN) acts as a molecular fertilizer in tissue culture, supplying a source of nitrates to the plants. Figure 3 shows that the application of CN increased the number of leaves for both genotypes (*p* < 0.005). The standard concentration of 556 mg/L seems to be sufficient to promote full plant growth and a higher concentration might be superfluous for the observed period.

### 2.3. Light Quality Optimization

**Figure 4:**
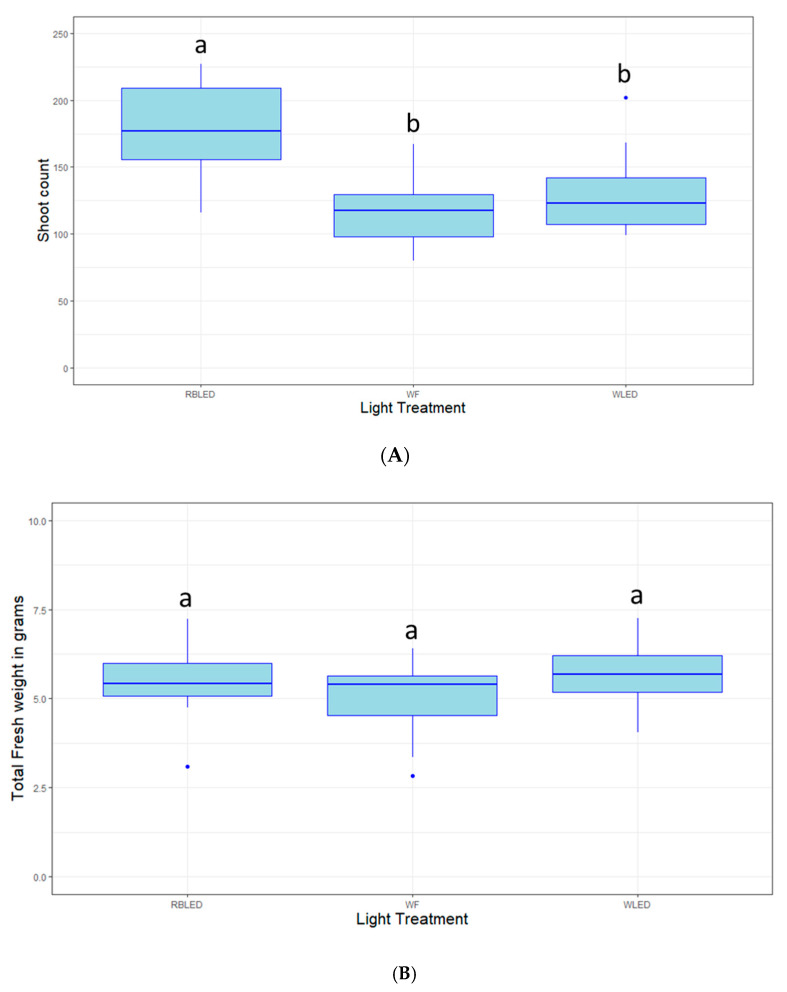

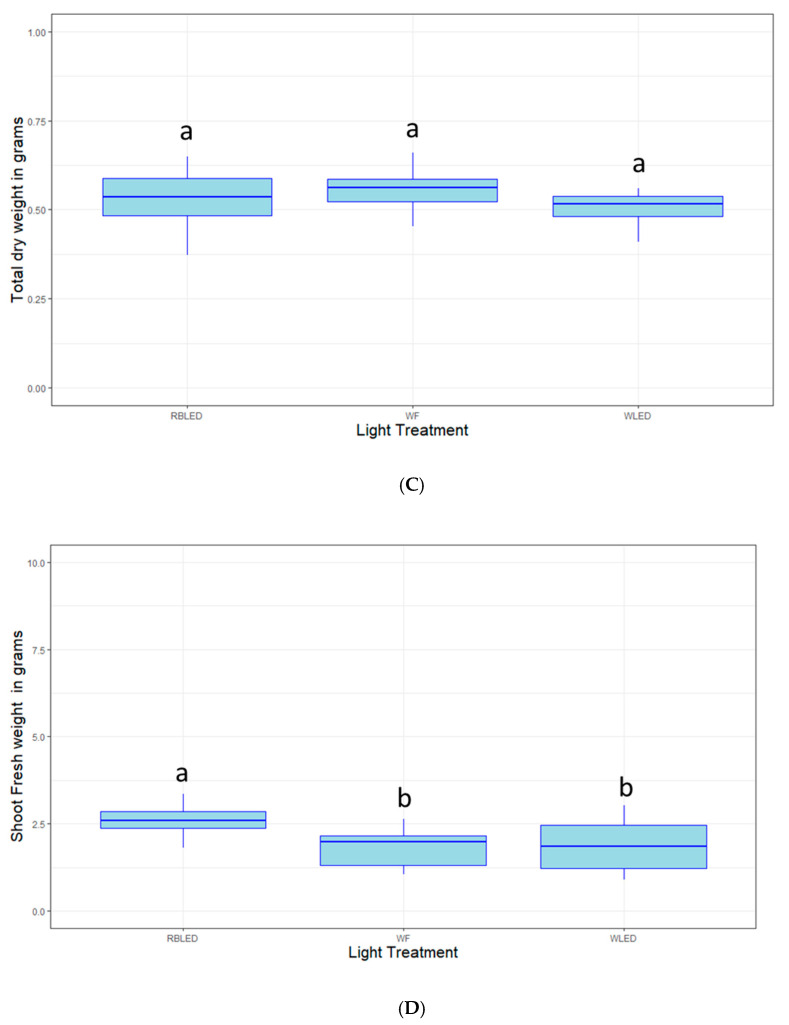

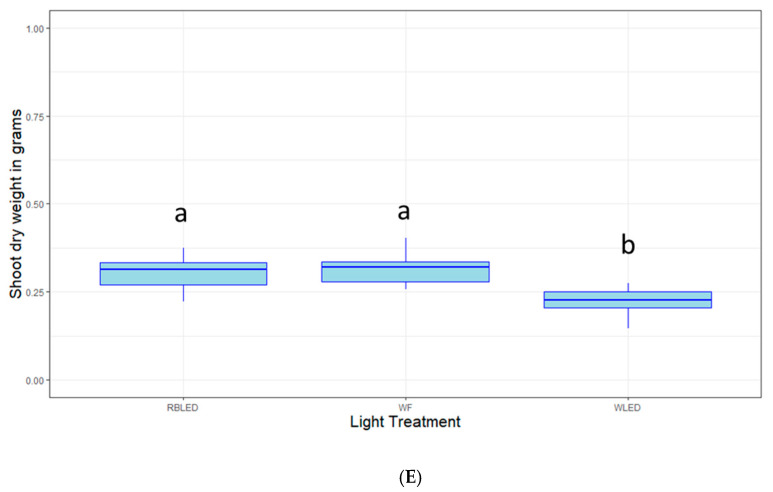
Box plot distribution of the effect of Red-Blue LED (RBLED), White Fluorescent (WF) and White LED (WLED) light sources on (**A**) shoot proliferation; (**B**) total fresh weight of callus and stems; (**C**) fresh weight of stems only; (**D**) dry weight of calli and stems; (**E**) dry weight of stems only. These experiments were carried out using the southern highbush blueberry cultivar “Farthing” grown for 100 days in stock culture media. Letters above boxplots indicate multiple comparison test. Treatments with the same letters are not statistically different at alpha = 0.05.

Light is not only a source of energy for photosynthesizing organisms but can also function as a hormone. In the case of blueberry plants in tissue culture, after the 30- and 60-day evaluation periods, the treatments showed no significant difference in shoot growth (*p* > 0.05, data not shown). After 100 days in treatment, plants grown under RBLED (Red Blue LED) lights produced significantly more shoots than those grown under WLED (White LED) or WF (White Fluorescent bulbs) (*p* < 0.01). Total (shoots + calli) fresh weight and dry weight measured after 100 days in treatment showed no significant difference between the treatments (*p* > 0.05). Likewise, when only taking into account the shoots (with the calli removed) plants grown under RBLED lights had significantly higher shoot fresh weight than those grown under WLED or WF (*p* < 0.01). In the case of dry weight, plants grown under WLED lights showed significantly less weight than those grown under RBLED and WF lights (*p* < 0.001). The effects of these three lighting conditions on various plant responses can be visualized in Figure 4.

### 2.4. Response to Decontamination Agents and Selectable Markers

**Figure 5:**
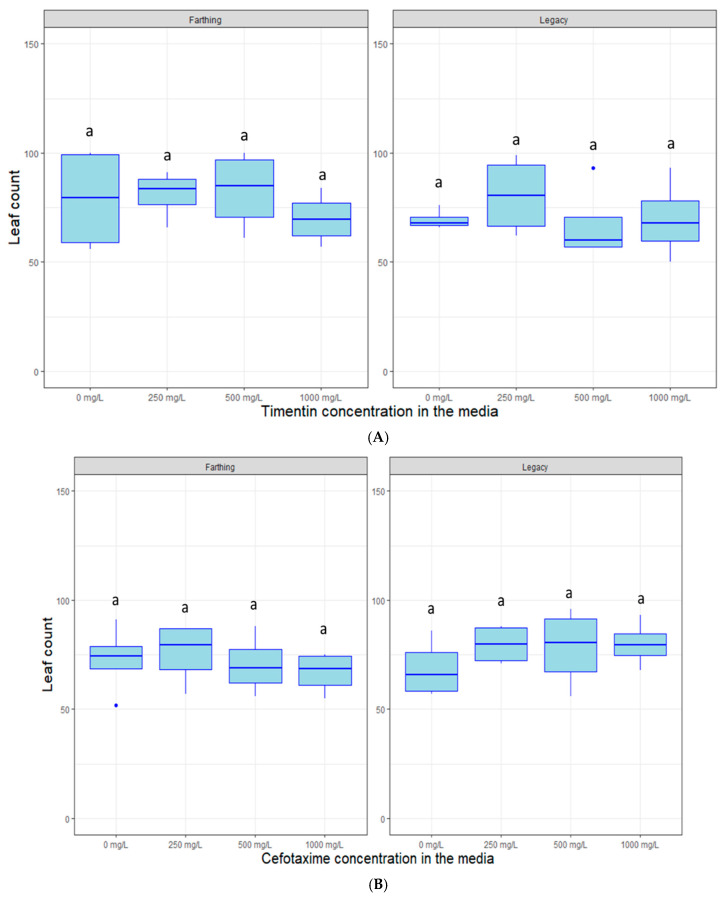
Box plot distribution of the effect of decontamination agents (**A**) timentin and (**B**) cefotaxime on leaf proliferation of blueberry genotypes “Farthing” (left panels) and “Legacy” (right panels) grown in tissue culture. Letters above boxplots indicate multiple comparison test. Treatments with the same letters are not statistically different at alpha = 0.05.

Cefotaxime and timentin are decontamination agents routinely used in plant transformation to remove *Agrobacterium* infections once the micro-organism has completed its DNA-transfer task. This is done to prevent over-growth of *Agrobacterium* which can lead to increased mortality and even completely take over the tissue culture space. Figure 5 highlights that there are no noticeable detrimental effects of the decontamination agents timentin (*p >* 0.05) or cefotaxime (*p >* 0.05) on plant growth, measured as leaf proliferation, at the different concentrations used.

**Figure 6:**
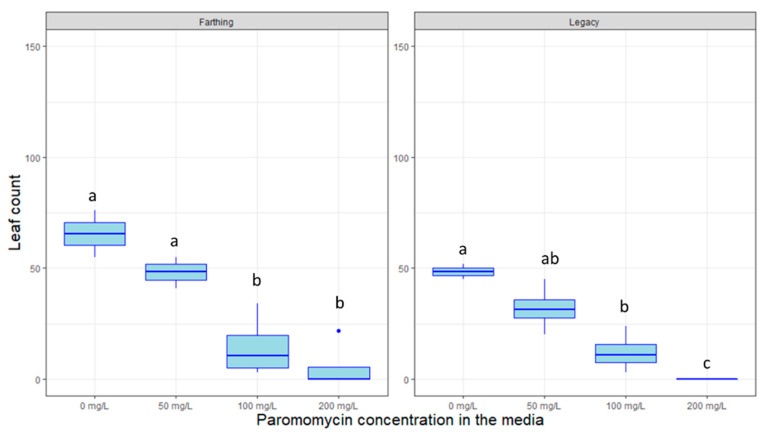
Box plot distribution of the effect of the selectable antibiotic marker kanamycin on leaf proliferation of two blueberry genotypes grown in tissue culture, “Farthing” (**left** panels) and “Legacy” (**right** panels). Letters above boxplots indicate multiple comparison test. Treatments with the same letters are not statistically different at alpha = 0.05.

**Figure 7:**
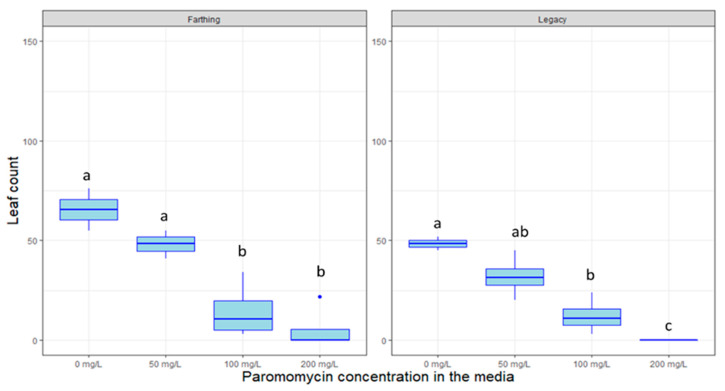
Box plot distribution of the effect of the selectable antibiotic markers paromomycin on leaf proliferation of two blueberry genotypes grown in tissue culture, “Farthing” (**left** panels) and “Legacy” (**right** panels). Letters above boxplots indicate multiple comparison test. Treatments with the same letters are not statistically different at alpha = 0.05.

**Figure 8:**
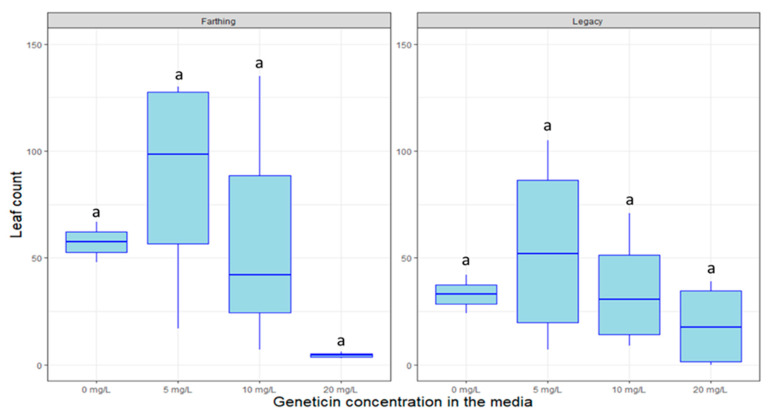
Box plot distribution of the effect of the herbicide glufosinate on leaf proliferation of blueberry genotypes “Colossus” (**left** panel) and “Legacy” (**right** panel). Letters above boxplots indicate multiple comparison test. Treatments with the same letters are not statistically different at alpha = 0.05.

Other antibiotics such as kanamycin are used to highlights plants that are successfully transformed by eliminating non-transformed plants that do not carry resistance genes. Figure 6, Figure 7 and Figure 8 display the inhibitory effects of kanamycin, paromomycin, and geneticin on the growth of genotypes in tissue culture. The genotype “Legacy” was susceptible to high doses of kanamycin (*p* < 0.005), geneticin (*p >* 0.05) and paromomycin (*p* < 0.01). Likewise, the “Farthing” genotype was inhibited by high doses of kanamycin (*p* < 0.005), geneticin (*p >* 0.05) and paromomycin (*p* < 0.05). For all selectable agents, the inhibitory effects are proportional to the antibiotic’s concentration. Surprisingly, an exception to this trend is that kanamycin at a concentration of 150 mg/L seems to almost promote plant growth in both genotypes.

**Figure 9:**
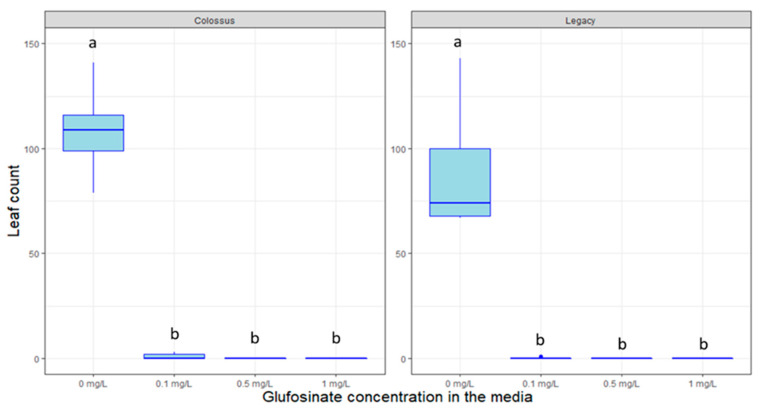
Box plot distribution of the effect of the herbicide glufosinate on leaf proliferation of blueberry genotypes “Colossus” (**left** panel) and “Legacy” (**right** panel). Letters above boxplots indicate multiple comparison test. Treatments with the same letters are not statistically different at alpha = 0.05.

Like in the case of antibiotics, herbicides such as glufosinate can also be used to select transformed plants. Figure 9 shows the significant effect of glufosinate on plant growth of the genotype “Colossus” (*p* < 0.005). The lower concentration completely inhibits plant growth. Please notice this test was performed on a different genotype than the other, the genotype “Colossus” was selected as it is more vigorous than “Legacy” and “Farthing”. In all treatment groups except for the control, glufosinate prevented the production of new plant growth in tissue culture. No statistically significant differences were detected within the 0.1, 0.5, and 1 mg/L treatment groups since no new plant growth occurred for any of them. Similar results were observed for higher concentrations of glufosinate (data not shown).

## 3. Discussion

Blueberry tissue culture is critical to both commercial plant propagation and research. Here we focus on tissue culture factors critical to propagation and transformation for a genetic subset of blueberry, LC blueberry varieties. The results of this research compare two representative LC blueberry varieties “Farthing” and “Colossus” to the HC blueberry variety “Legacy” which has been traditionally used for several tissue culture applications [18].

In this study, we elaborated a novel set of practices (referred to as the advanced technique) aimed at increasing the survival rate of LC blueberry plants introduced to tissue culture from field conditions. The basic technique was taken from Cüce et al. [37] and adapted to LC blueberry using insights from the work of Sathyanarayana and Verghese (2007) [38], and Smith (2012) [39]. It should be noted that the protocol reported here can serve as a general guideline and will most likely be effective for the majority of LC blueberry genotypes. However, varying the ethanol/bleach exposure could yield better results depending on the genotype that is being introduced to tissue culture conditions.

In *Vaccinium*, CN is used both as a fertilizer when added to the medium [4,18], and as a microbicide and fruit quality enhancer, when used as a foliar spray [40,41]. From a media composition perspective, the results presented here are in line with previous literature; suggesting that 556 mg/L of CN are optimal for blueberry growth [4,18]. Zeatin is a hormone routinely used in blueberry tissue culture [8,9,11,12], which has been shown to outperform 2iP [8,10]. Our results focus on zeatin and indicate that for the genotype “Farthing” (Error! Reference source not found.) and “Colossus”, representative of LC growth habits, concentrations in the 1–2 mg/L range are optimal to promote the growth of plants best suited for transformation, i.e., with few meristems and few large leaves. The same zeatin concentration was also found to be favorable to the growth of “Legacy”, representative of the HC genetic backgrounds. Higher zeatin concentrations increased the amounts of leaves produces, but at the cost of leaf size, which was often observed to be <1 mm. This is also in line with previous findings [8,9,11,12]. Light, which can also be regarded as a growth regulator, was shown to moderately affect plant growth. The red-blue LED lights yielded more shoots and more biomass when compared to the white fluorescent and white LED lights. Our meristem count results were in line with previous findings in blueberry [42]. However, the same study also reported that RB LEDs were leading to increased biomass when compared to white fluorescent bulbs over a 6-week period, while we found that light had no effect on the biomass over a 100-day period [42]. Slightly different results were also reported in grape, a genetically similar crop, for which RB LEDs did not significantly affect shoot number or biomass when compared to white fluorescent bulbs [43]. Overall, our results suggest that red-blue LED lights might be suitable for commercial operations that require numerous meristems and biomass that are used to propagate genotypes at a sustained pace. For transformation operations, white fluorescent lights might still be more suitable as the lower number of meristems leads to larger, easier to handle, leaves. Future endeavors could focus on optimizing light conditions for newly adopted genotypes and longer growth periods. In addition, more research could explore the use of light to enhance production of anthocyanins and other compounds that make blueberries attractive to consumers [44,45]. Light quality has been shown to affect anthocyanin production in blueberry and other crops, even though this phenomenon seems to be more related to UV light, than light in the visible spectrum [46,47,48].

This research also shows that very high levels of the decontamination agents timentin and cefotaxime can be used to remove *Agrobacterium* overgrowth without affecting the plants. This is especially useful when employing the hyper-virulent EHA105 strain, which is the best-suited strain to transform blueberry. Previously published research indicates that amounts as low as 250 mg/L are routinely used to control *Agrobacterium* overgrowth [18]. However, the results presented here show that concentrations as high as 1000 mg/L do not inhibit plant growth and effectively suppress most gram-negative contaminations. This increased concentration might allow for better micro-organism control and can allow for better removal of bacteria dwelling deeply in the plant tissue. This is especially relevant in the later transformation stages when the total sample DNA is extracted, and the presence of transgenes is verified via PCR markers, where residual transgene-carrying bacterial DNA can cause false positives.

In partial contrast to what has been reported in the literature, our findings do not support the use of kanamycin, geneticin, or paromomycin as selectable markers in blueberry, neither for the HC genotype “Legacy” nor for the LC genotype “Farthing”. Kanamycin was reportedly able to inhibit “Legacy” growth at 10 mg/L [4,18]. Here, we show that significant growth inhibition was only achieved at very high doses; 300–400 mg/L in the case of Kanamycin. Such high doses are not advisable as they might cause unintended effects or could lead to false-positive visual marker results due to incomplete selection. It can be speculated that tolerance to the abovementioned antibiotics could be highly genotype-specific. This is especially relevant in LC blueberry, where certain genotypes have been created crossing northern highbush blueberry with very different southern-adapted *Vaccinium* species [36]. Future efforts could comprehensively test selection tolerance in a wide range of LC blueberry cultivars with varied pedigrees in an attempt to discern whether one of the ancestral species provides a source of tolerance to antibiotic selection.

On the other hand, the herbicide glufosinate, shows promise to be a considerably more efficient selectable marker in blueberry, as previously shown by Song (et al. 2005, 2008) [22,49]. Even at extremely low dosages (0.1 mg/L), glufosinate can completely inhibit blueberry plant growth. All plants exposed to glufosinate in our study suffered lethal damage from the exposure, rendering them incapable of producing new leaves or stems. Currently, studies are taking place to confirm glufosinate as a selectable marker by assessing the survivability of genetically transformed blueberry plants in vitro.

The research presented here represents a crucial step in advancing tissue culture tools for LC blueberry. In particular, this research provides the foundation for future transformation experiments in this crop. Most plant transformation systems are based on kanamycin resistance. Knowing that this antibiotic (and similar ones) are not viable in LC blueberry could save months-worth of research to future transformation endeavors, which should favor herbicide markers such as glufosinate.

Future research should clarify the optimal amount of glufosinate in the media to select plants that carry the resistance transgene and also explore the role of fluorescent and visual transformation markers in selected modern genotypes of LC blueberry. Besides, a new class of tools could be employed to rapidly optimize tissue culture conditions for emerging crops like blueberry once a critical mass of data is gathered. In silico predictive deep learning algorithms such as artificial neural networks can process large and diverse datasets and provide high-throughput solutions [50,51]. These techniques have been utilized to optimize regeneration in wheat [52], *Chrysanthemum* spp. L. [53,54], *Swertia paniculata* (Wall.) [55], *Bryophyllum* (Salisb.) [56], *Prunus* spp. L. [57,58], and *Cucurbitaceae* L. [59]. Large-scale studies in blueberry could also greatly benefit from a design that allows the use of these techniques.

## 4. Materials and Methods

### 4.1. Plant Genotypes and Growth Conditions

Blueberry plants were grown under standard field conditions in Waldo, FL (USA). Non-lignified stems measuring 10–20 cm were excised from healthy plants actively growing. Explants were collected from the genotypes “Farthing” “Legacy” and “FL11-35” (also known as Colossus). Leaves were removed from the stems to reduce fungal and bacterial loads and the stems were cut into sections 3–6 cm long, containing two to eight nodes each. Plants were grown under 16 h/8 h fluorescent lighting at room temperature for 4–6 weeks. In the case of plants that were sub-cultured, after new shoots began to emerge, plants were cut into two-node segments and relocated to fresh media.

### 4.2. Introduction to Tissue Culture from Field Conditions

A basic technique was initially adopted based on the works of Cüce et al. [37], and then refined in an advanced technique, both described below. Elaboration of the advanced technique was the result of numerous attempts that altered one aspect of the process at a time. Many of such attempts were not successful, so here we just report the comparison between the initial technique i.e., the basic technique, and the final set of practices that we adopted, i.e., the advanced technique. These basic and advanced techniques were tested in a randomized complete block design with five replicates and 30 explants in each replicate. Mortality rates were recorded for both techniques across the genotypes “Legacy” and “Farthing”. The percentage includes both plant cultures contaminated by microorganisms and explants that did not survive the chemical treatment.

In the basic technique, the stems were washed in cold, running tap water for six minutes, then moved to the laminar flow hood, where they were rinsed in 70% ethanol for one minute. Next, the plant cuttings were agitated in a 10% Clorox solution for 10 min, rinsed with sterile water for one minute, dried with a sterile paper tissue, and finally placed in stock culture media. In the advanced technique, the stem segments were washed in cold, running tap water for six minutes, washed by hand with antibacterial soap, and rinsed in running tap water for an additional six minutes. Stems were then moved to a laminar flow hood, where they were rinsed in 70% (*v/v*) ethanol for one minute. They were transferred to a 10% bleach (*v/v*) solution for 6 min before being rinsed in sterile water 5 times for 1 min each time. During the final rinsing step, stems were cut into small segments by removing the top and bottom petiole nodes and including only 1 node per segment. Stem cuttings were dried on autoclaved paper tissue before transferring to Stock Culture Medium (SCM).

This media to grow the explants was adapted from Song and Skin 2006 [4]. In brief, ingredients listed under Stock Culture Media were added to 1 L of de-ionized water and mixed. The pH of the solution was subsequently adjusted using 1 M KOH and/or 1M HCL until a desired final pH of 5.2 was reached. After pH adjustment, the solution was then autoclaved for 30 min. Zeatin was filter-sterilized (0.22 µm Millipore filters; Millipore Corporation, Bedford, MA, USA) and then added to the solution under sterile conditions in a quantity dependent on the genotype. If not otherwise indicated, the standard zeatin concentration was 2 mg/L.

### 4.3. Hormonal Dosage and Nutrient Optimization

Hormonal optimization allows for ideal leaf/plant architecture for downstream applications. SCM was supplemented with various amounts of the cytokinin, zeatin, in quantities of 0, 1, 3, and 5 mg/L. The nutrient calcium nitrate [Ca(NO_3_)2H_2_O] is used in tissue culture as a fertilizer. The published protocol by Song and Sink 2006 [4] suggested a standard concentration of 556 mg/L. For this experiment, calcium nitrate was tested by adding it to SCM (without any calcium nitrate) at the following concentrations: 0, 278, 556, and 1112 mg/L.

For both experiments, two-node plant cuttings were segmented from established cultures of “Farthing” and “Legacy” and introduced to the media. Each treatment consisted of four tissue culture vessels per variety, each with nine plant cuttings per container. Plants were grown for two months, under 16 h/8 h LED lighting at room temperature. Following the growth period, total leaf numbers were counted. For the hormonal growth experiment, the number of new shoots was also recorded, as zeatin is known to affect this trait.

### 4.4. Light Quality Optimization

To determine optimal lighting in tissue culture for genetic transformation, clonal plants from “Farthing”, were cut into three-node segments. The leaves were removed from the plants, and the stems were introduced into SCM.

The plants were then separated into three treatments containing 126 cuttings each. Treatment 1 (RBLED) was introduced to Red/Blue (77%/23%) light from Light-Emitting Diodes (LEDs), treatment 2 (WLED) was introduced to white light from LEDs, and treatment 3 (WF) was introduced to white light from fluorescent lighting. The treatments were incubated 25 ± 2 °C and were subjected to a light intensity of 55 ± 12 µmol m^−2^ s^−1^ for 12 h per day. The plants remained in treatment for 100 days, being evaluated at 30 and 60 days for shoot development.

At the end of the 100-day period in treatment, the plants were removed from the tissue culture vessels and phenotyped. The traits evaluated were: number of shoots produced, total (calli + stems) fresh weight, total dry weight, fresh weight without calli, and dry weight without calli.

### 4.5. Response to Selectable Markers and Decontamination Agents

Timentin and cefotaxime are antibiotics that are routinely used as decontamination agents to remove excess *Agrobacterium* growth in plant tissue culture. Both were tested on untransformed (“Legacy” and “Farthing”) SHB plants in tissue culture to determine whether these antibiotics negatively impact plant growth at high concentrations. Cefotaxime and timentin were filter sterilized and added to SCM at concentrations of 0, 250, 500, and 1000 mg/L.

Antibiotic resistance genes can serve as visual selectable markers of transformation success when plants are exposed to these antibiotics post-transformation. The antibiotics kanamycin, paromomycin, and geneticin were tested on untransformed SHB plants in vitro. All antibiotic solutions were filter sterilized (0.22 µm Millipore filters; Millipore Corporation, Bedford, MA, USA), and added to media cooled to 50–60 °C after autoclaving. Kanamycin was introduced in the media at concentrations of 0, 150, 300, 400 mg/L; paromomycin was introduced at 0, 50, 100, and 200 mg/L; and geneticin was introduced at 0, 5, 10, and 20 mg/L.

For kanamycin, 2-node segmented plant cuttings were taken from previously established “Legacy” and “Farthing” and introduced to new media. For each treatment, 4 boxes were used per treatment, with 9 plant cuttings in each box. For geneticin and paromomycin, 2-node segmented plant cuttings were taken from previously established SHB “Farthing” and “Legacy” and introduced to this new media. In the experiment, 2 tissue culture vessels were utilized per treatment, per variety, each with 9 plant cuttings. Plants were grown for 3 months in tissue culture, under 16 h/8 h LED lighting at room temperature. Data points for total leaf and shoot quantities were enumerated for each container at the one-month and three-month marks.

Another selectable marker, the herbicide glufosinate, was also tested on untransformed SHB plants in tissue culture to determine if a glufosinate resistance gene could serve as a good visual marker of successful transformation. Glufosinate was filter sterilized and introduced to SCM media at concentrations of 0, 0.1, 0.5, and 1 mg/L. Two-node segmented plant cuttings were taken from “Colossus” (LC) and “Legacy” (HC) and introduced to this new media. Four tissue culture containers were utilized per treatment, each with 9 plant cuttings per container. Plants were grown for two months in tissue culture, under 16 h/8 h LED lighting at room temperature. Following the two-month growth period, the total number of new stems was counted for each container.

### 4.6. Statistical Analysis

All experiments were established in a complete randomized design with three repetitions and two replicas starting October 2019 and March 2020, respectively. Each box (repetition unit) contained nine explants. Statistical analyses were carried out in the software R statistics [60]. ANOVA with Tukey post-hoc tests were used to highlight significant differences among biological treatments. Outliers were corrected for using the Interquartile Range Rule. Graphs were elaborated using the ggplot2 package of R [61].

## 5. Conclusions

Tissue culture for low-chill blueberry can be time-consuming. In addition, pushing a plant architecture suitable to plant transformation, i.e., few large leaves, can be challenging. In this paper, we demonstrated that low amounts of zeatin (1–2 mg) and white fluorescent light sources are best for the abovementioned purpose. Also, the evidence presented here suggests that kanamycin, paramomycin, and genetic are not effective markers to select transformed plants, whereas glufosinate is an effective one.
